# Efficiency Enhancement of Dye-Sensitized Solar Cells’ Performance with ZnO Nanorods Grown by Low-Temperature Hydrothermal Reaction

**DOI:** 10.3390/ma8125499

**Published:** 2015-12-19

**Authors:** Fang-I Lai, Jui-Fu Yang, Shou-Yi Kuo

**Affiliations:** 1 Department of Photonics Engineering, Yuan-Ze University, 135 Yuan-Tung Road, Chung-Li 32003, Taiwan; filai@saturn.yzu.edu.tw (F.L.); yoharecca@gmail.com (J.Y.); 2 Advanced Optoelectronic Technology Center, National Cheng-Kung University, Tainan 70101, Taiwan; 3 Department of Electronic Engineering, Chang Gung University, 259 Wen-Hwa 1st Road, Tao-Yuan 33302, Taiwan; 4 Department of Green Technology Research Center, Chang Gung University, 259 Wen-Hwa 1st Road, Tao-Yuan 33302, Taiwan

**Keywords:** dye-sensitized solar cells, nanorods, AZO film

## Abstract

In this study, aligned zinc oxide (ZnO) nanorods (NRs) with various lengths (1.5–5 µm) were deposited on ZnO:Al (AZO)-coated glass substrates by using a solution phase deposition method; these NRs were prepared for application as working electrodes to increase the photovoltaic conversion efficiency of solar cells. The results were observed in detail by using X-ray diffraction, field-emission scanning electron microscopy, UV-visible spectrophotometry, electrochemical impedance spectroscopy, incident photo-to-current conversion efficiency, and solar simulation. The results indicated that when the lengths of the ZnO NRs increased, the adsorption of D-719 dyes through the ZnO NRs increased along with enhancing the short-circuit photocurrent and open-circuit voltage of the cell. An optimal power conversion efficiency of 0.64% was obtained in a dye-sensitized solar cell (DSSC) containing the ZnO NR with a length of 5 µm. The objective of this study was to facilitate the development of a ZnO-based DSSC.

## 1. Introduction

Dye-sensitized solar cells (DSSC) belong to the third generation of solar cells. Due their low-cost materials and low-cost technologies, they are the promising replacement for conventional silicon-based solar cells [1]. The highest single-cell conversion efficiency of 13% is comparable to the Si cells [2]. Generally, TiO_2_ nanoparticle films coated onto fluorine-doped tin oxide (FTO) layers are made as the photoelectrode in DSSCs because of their suitable chemical affinity and surface area for dye adsorption as well as their proper energy band promising charge transfer between the electrolytes and dye [3,4]. However, the one problem of DSSCs is that not all of the photogenerated electrons can arrive at the collecting electrode, because electron transport within the nanoparticle network takes place via a series of hops to adjacent particles, and the energy damage that occurs during charge transport processes results in conversion efficiency. This trapping process results in the transport becoming slow, and an increase in scattering, which greatly increases the recombination of the electrons with the oxidized dye molecules, reducing efficiency and oxidized redox species. In order to enhance dye adsorption, the thickness of TiO_2_ should be increased. However, this recombination problem is aggravated in TiO_2_ nanocrystals by reason of a depletion layer on the TiO_2_ nanocrystallite surface, and its severity increases as the photoelectrode film thickness increases [5]. In response to this problem, the paper proposes a ZnO-based DSSC technology as a replacement for TiO_2_ in solar cells. Zinc oxide has received a great deal of attention as a photoanode in dye-sensitized solar cells (DSSCs) due to its large exciton-binding energy (60 meV) and large band gap (3.37 eV) [6]. Furthermore, its electron mobility is higher than that of TiO_2_ by two-to-three orders of magnitude [7]. Therefore, ZnO is anticipated to demonstrate faster electron transport as well as decreased recombination damage compared to TiO_2_. Nevertheless, studies have reported that the entire efficiency of TiO_2_ DSSCs is higher than that of ZnO DSSCs. The efficiency of TiO_2_ thin-passivation shell layers is higher than the highest reported efficiency of ZnO DSSCs [8], in which the principal problem is the dye adsorption process in ZnO DSSCs. Because of the high carboxylic acid binding groups in the dyes, the dissolution of ZnO and precipitation of dye-Zn^2+^ complexes occurs. This phenomenon results in a poor overall electron injection efficiency of the dye [9].

Several approaches exist for enhancing the efficiency of ZnO DSSCs. One method is to introduce a surface passivation layer to a mesoporous ZnO framework; nevertheless, this may aggravate the dye adsorption problems. Alternatively, conventional particulate structures can be changed by replacing the internal surface area and morphology of the photoanode. Nevertheless, the surface area and diffusion length are incompatible. Augmenting the photoanode thickness empowered a higher number of dye molecules to be fixed; this, however, increases the possibility of electron recombination because of the extended distance through which electrons diffuse to the transparent conductive oxide (TCO) collector. This trapping process results in augmented scattering and slows down the electron transport which increases the recombination of the electrons with the oxidized redox species or the oxidized dye molecules, hence reducing efficiency. One probable strategy for ameliorating electron transport in DSSCs is to supersede the nanoparticle photoelectrode with a single-crystalline nanorod (or nanosheet, nanobelt, nanotip) photoelectrode. Electrons can be led through a direct electron path within a nanorod rather than by multiple-scattering transport between nanoparticles. In research, the electron transport is tens to hundreds of times slower in nanoparticle DSSCs than in nanorod-based DSSCs [10,11,12]. Therefore, many works have been performed on the synthesis of TiO, and ZnO nanostructures for applications in DSSCs [13,14,15].

However, the utilization of FTO may not be the best method for improving the cell performance. One problem is that the small difference in the work function between ZnO and FTO does not supply sufficient driving force for the charge injection from the ZnO nanowires to FTO, which hints that new TCO materials should be used in ZnO-based DSSCs. Lee *et al.* use the ZnO:Al (AZO) film to replace the FTO layer as the TCO layer [4]. Their structure was accomplished by a three-step process, TCO, seed layer, and nanostructure, but this method was slight complicated. To simplify the procedures, we used a two-step process in this study, and present a detailed discussion. These characteristics were observed using X-ray diffraction (XRD), UV-visible spectrophotometry, field-emission scanning electron microscopy (FE-SEM), electrochemical impedance spectroscopy (EIS), incident photon-to-electron conversion efficiency (IPCE), and solar simulation.

## 2. Experimental

**Figure 1:**
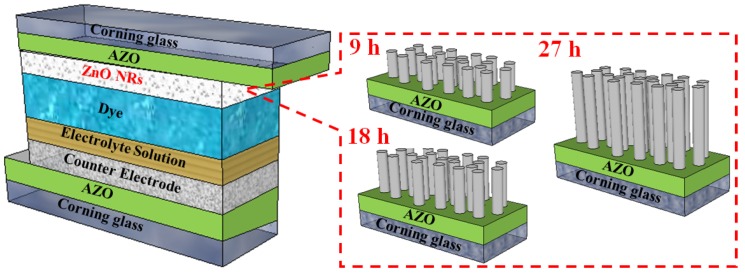
The schematic illustrations of DSSCs with ZnO nanorods.

Figure 1 illustrates the schematic structures of DSSCs with ZnO nanorods of various lengths, which are shown in Figure 1. First, radio-frequency sputtering was used to deposit a ZnO:Al (AZO) seed layer (approximately 300 nm) on Corning-glass substrates with a sheet resistance of 18 Ω/sq, and the defined area of the seed layer was 1 cm^2^. The Pt (H_2_PtC_l6_ solid content: <6%, viscosity: ~50 cps, eversolar Pt-100) film was also deposited on the AZO/Corning-glass substrates by spin-coating. These substrates were used for growing ZnO NRs. The ZnO NRs were deposited using zinc nitrate (Zn(NO_3_)_2_6H_2_O, Aldrich) and hexamethylenetetrasece (C_6_H_12_N_4_, HMT, Aldrich). Both mixtures were melted in deionized water to a concentration of 0.02 M and stored at 90 °C for 9 h. These solutions were replaced every 9 h, and the corresponding ZnO NRs were denoted by 18- and 27-h NRs. The hydrothermal chemical reactions for the ZnO NRs are expressed as follows:

*C*_6_*H*_12_*N*_4_ + 6*H*_2_*O* → 6*HCHO* + 4*NH*_3_ (1)

*NH*_3_ + *H*_2_*O* → *NH*_4_^+^+ *OH*^−^ (2)

*Zn*^2+^+2*OH*^−^→ *Zn*(*OH*)_2_ (3)

(4) $$Zn{\left(OH\right)}_{2}\;\underset{\mathrm{heat}}{\to }\;ZnO\;+\;H_{2}O$$

After the reaction was complete, the resulting ZnO NRs were rinsed with deionized water to remove residual ZnO particles and impurities. A D-719 dye, *cis*-bis(isothiocyanato)bis(2,2′-bipyridyl-4,4′-dicarboxylato) ruthenium(II)bis-tetrabutylammonium, (Everlight Chemical Industrial Corp., Taipei, Taiwan) was dissolved in acetonitrile for preparing a 0.5 mM dye solution. Dye sensitization was propagated by soaking the ZnO photoelectrodes in the D-719 dye at room temperature for 2 h. A sandwich-type configuration was used to measure the presentation of the DSSCs. An active area of 1 cm^2^ was assembled by using a Pt-coated AZO substrate as a counter electrode, and the Pt/AZO was heated at 200 °C for 30 min in air. The DSSC was sealed employing a polymer resin (Surlyn) to act as a spacer. The electrolyte was injected into the space among the electrodes from these two holes, and then these two holes were sealed completely by using Surlyn. The electrolyte (0.5 M 4-tert-butyl-pyridine + 0.05 M I_2_ + 0.5 M LiI + 0.6 M tetrabutylammonium iodide) was injected to the cell and then sealed with UV gel. The influence of growth time on the structural and optical properties of these ZnO NRs was analyzed by XRD and UV-visible spectrophotometry. Surface morphologies of the ZnO nanorods were examined using field-emission scanning electron microscope (FESEM). The photocurrent-voltage (I-V) characteristic curves were measured using Keithley 2420 under AM 1.5 illumination. The electrochemical impedance spectroscopy (EIS) was measured under the light illumination of AM 1.5 G (100 mW/cm^2^) with an impedance analyzer (Autolab PGSTAT 30) (Metrohm Autolab, Utrecht, Netherlands) when a device was applied with its open-circuit voltage (Voc). An additional alternative sinusoidal voltage amplitude 10 mV was also applied between an anode and a cathode of a device over the frequency range of 0.02~100 kHz. The external quantum efficiency (EQE) results were acquired from a system using a 300 W xenon lamp (Newport 66984) light source and a monochromator (Newport 74112) (Newport Corporation, Taipei, Taiwan). The beam spot size at the sample measured was approximately 1 mm × 3 mm. The temperature was controlled at 25 °C during the measurements.

## 3. Results and Discussion

**Figure 2:**
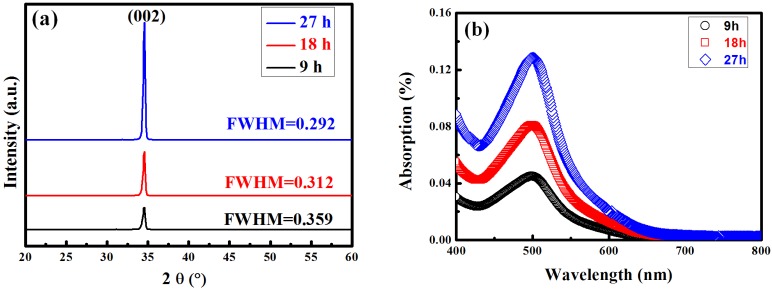
(**a**) XRD patterns of ZnO nanorods grown with different duration; (**b**) Optical absorption spectra of D-719 dye detached from the ZnO NRs with various lengths.

In this study, ZnO NRs with various lengths were grown on AZO substrates of photoanodes to increase the optical absorption of the dye. Figure 2a shows the respective XRD patterns for the ZnO NRs derived from the 9-, 18-, and 27-h reactions, respectively. The crystalline structure was analyzed using XRD measurements according to a θ/2θ configuration. In principle, the XRD spectra indicate that the ZnO films developed without the presence of secondary phases or groups. All the samples have a hexagonal wurtzite structure of ZnO and grew along the c-axis; this enabled the observation of the ZnO (002) diffraction plane in the XRD pattern. The increase in intensity of the diffraction peak and also the narrowing of the peak, in other words, decrease in the full width at half maximum (FWHM) of the peak, with the length of ZnO NRs increased, and the crystallinity improvement of the ZnO NRs. Existing dye uptake measurements were based on dye desorption from the photoanode after a specified 30 min using a NaOH solution, and the succeeding UV-Vis spectroscopy. For the quantitative analysis of dye loading, the washing course for desorption of dye from the anodes was performed using the known volume of 0.1 mM NaOH aqueous solution. The dye detached from the ZnO NRs as implemented for different lengths of ZnO NRs in literature. Figure 2b illustrates the absorptions of solutions containing 0.01 mM dye, indicating that dyes detached from the ZnO NRs at 9- (black line), 18- (red line), and 27-h (blue line), respectively. The area of both films was 1 cm^2^. The results depicted in Figure 2b can be used to calculate the dye loadings and light absorptions at 530 nm (the peak dye absorption) for the ZnO NRs from the 9-, 18-, and 27-h reactions. The lengths of the 18- and 27-h ZnO NRs were longer than those of the 9-h ZnO NRs, and they demonstrated an improvement in light harvesting and dye loading with increased NRs.

**Figure 3:**
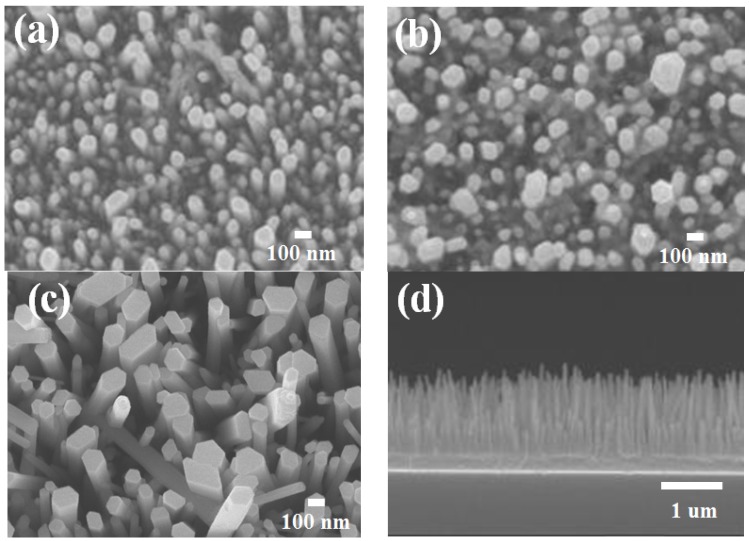

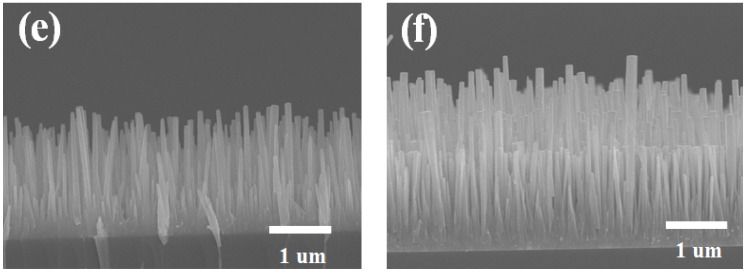
SEM images of ZnO nanorods fabricated under various growth time. (**a**–**c**) Top-view images of ZnO nanorods grown at 9 h, 18 h, and 27 h; (**d**–**f**) Side-view images of ZnO nanorods grown at 9 h, 18 h, and 27 h, respectively.

As mentioned, ZnO NRs with various lengths were grown on AZO substrates, and these NRs were used in DSSCs (Figure 3). Figure 3a–f illustrate FE-SEM images of the respective ZnO NRs from the 9-, 18-, and 27-h reactions grown on the AZO substrates, indicating that the ZnO NRs were adequately grown on substrates with a distinctive, clear morphology. Furthermore, the diameters, lengths, and aspect ratios of the NRs were in the range of 76–110 nm, 1.5–5 μm, and 20.7–47.9, respectively. Greene *et al.* indicated that the growing temperature influences the upright growth of ZnO NRs [16].

**Figure 4:**
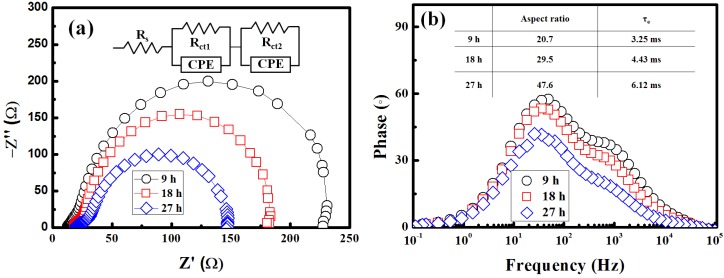
Electrochemical impedance spectra of DSSCs containing ZnO nanorods with various lengths. (**a**) Nyquist plots and (**b**) Bode phase plots. The equivalent circuit of this study is shown in the inset of (**a**).

Figure 4a depicts the Nyquist plots of the impedance spectra. To characterize the AZO/dye/electrolyte interface, the open-circuit voltage (Voc) levels of the DSSCs were evaluated under AM 1.5 illumination by conducting EIS measurements. The Nyquist plots indicate a small semicircle at high frequencies and a large semicircle at low frequencies. The inset in Figure 4a shows the equivalent circuit. Usually, all the spectra of the DSSCs exhibit three semicircles, which are ascribed to the electrochemical reaction at the Pt counter electrode, charge transfer at the TiO_2_/dye/electrolyte, and Warburg diffusion process of I^−^/I^3−^, respectively [17,18]. In the present study, the charge transfer resistance at the ZnO/dye/electrolyte interface (Rct_2_) decreased when the aspect ratio of the ZnO NRs was varied from 20.7 to 47.6. This may be attributable to the increase in the diameter size, length, and quality of ZnO NRs, which led to an increase in the dye adsorption as well as penetration of electron mobility into the pores of the AZO electrode (Figure 4a). The better collected and transported electrons had a lower possibility of recombination, and the electron lifetime was increased [19]. Figure 4b shows Bode phase plots indicating the characteristic frequency peaks (1–10^4^ Hz). The characteristic frequency peak shifted to a lower frequency when the aspect ratio increased, and the characteristic frequency can be considered as the inverse of the electron lifetime (*τ_e_*) or recombination lifetime (*τ_r_*) in an AZO film [20,21]. This implies that the NRs with an aspect ratio of 47.6 (grown for 27 h) had the longest electron lifetime in the AZO film. The results indicate that the ZnO NRs, which were grown for 27 h (aspect ratio: 47.6), on the AZO film had a lower transport resistance and a longer electron lifetime in the AZO electrode. The electron lifetimes in the AZO films increased from 3.25 to 6.12 ms when the aspect ratio increased from 20.7 to 47.6. This result is consistent with the following results obtained from cell performance and EIS analysis.

**Figure 5:**
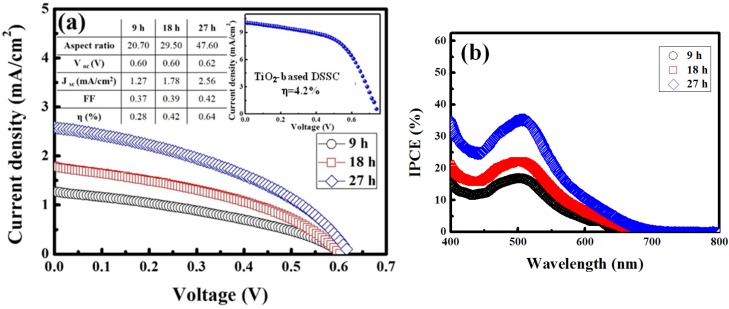
(**a**) J-V measurements under AM 1.5 illumination (100 mA·cm^−2^ ) and (**b**) IPCE spectra of DSSCs containing ZnO nanorods grown at various durations. Shown in the inset of Figure 5a is the photovoltaic performance of DSSC employing TiO_2_ nanoparticles.

Figure 5a shows the J-V curve for the DSSCs containing the ZnO NRs obtained from the 9-, 18-, and 27-h reactions, indicating that the short-circuit current density (Jsc) and cell performance significantly increase with the NR length. As revealed in the figures, the photovoltaic performances of our DSSCs employing ZnO NRs are comparable to published literature [22,23,24]. A higher amount of dye was adsorbed on longer NRs than on shorter NRs, indicating that longer NRs improve photon absorption and carrier generation. These results indicate that cell performance is strongly dependent on the electrode surface area. Increasing the NR length results in a larger surface area, which leads to a higher adsorption of dyes as well as a higher conversion efficiency. Furthermore, the Voc of the longer ZnO NRs was higher than that of the shorter ZnO NRs. This higher Voc is attributable to a reduction in recombination losses at ZnO/dye interfaces. Regarding the performance of the cells containing the ZnO NRs grown for various periods, the cell containing the 27-h ZnO NRs demonstrated optimal performance with a conversion efficiency (η) of 0.64%, Voc of 0.62 V, Jsc of 2.56 mA/cm^2^, and fill factor of 0.42. The NRs also provide direct pathways from the point of photogeneration to the conducting substrate. These pathways ensure the rapid collection of carriers generated throughout the device. Figure 5b depicts the IPCE spectra of the DSSCs (D-719 dye) containing the 9-, 18-, and 27-h ZnO NRs, indicating a strong peak at 520 nm; this peak is attributable to the characteristic excitations of the D-719 dye. Our ZnO-based DSSCs show poor conversion efficiencies when compared to conventional TiO_2_-based DSSCs, as shown in the inset of Figure 5a. The main reason is the corrosion of ZnO on reacting with an acid and the low amounts of dyes that are adsorbed during the production. During the process, an amount of Zn^2+^ ions are dissolved into the solution from the surface of the ZnO nanorods. Subsequently, aggregation of Zn^2+^ ions with sensitizer dyes occurs, and the phenomenon was reported for several organic sensitizer dyes as well as ruthenium complexes [25,26]. Once aggregation takes place in DSSCs, the power conversion efficiency will dramatically decrease [27]. Despite the lower efficiencies in our ZnO-based DSSCs, the use of ZnO nanorods still shows high potential because of its better crystallinity and higher electron mobility. To overcome the chemical instability of ZnO, the introduction of non-ruthenium-based sensitizers and the utilization of different nanotechnological architectures of ZnO might be practical approaches.

## 4. Conclusions

In this study, we prepared ZnO NRs, with a two-step process which is simple and easy, for use as photoanodes in DSSCs. Moreover, the results reveal that DSSCs containing longer ZnO NRs demonstrate higher photovoltaic performance than DSSCs containing shorter ZnO NRs. Compared with shorter ZnO NRs, longer ZnO NRs exhibit a larger surface area, which enables efficient dye loading and light harvesting, reduced charge recombination, and faster electron transport. These improvements enhanced power conversion for application in DSSCs.
